# Primary MRSA Myositis Mimicking Septic Arthritis

**DOI:** 10.1155/2023/5623876

**Published:** 2023-02-28

**Authors:** Mohammad Abu-Abaa, Amy Pulikeyil, Hassaan Arshad, Daniel Goldsmith

**Affiliations:** Capital Health Regional Medical Center, Internal Medicine Residency Program, USA

## Abstract

As the incidence of bacterial myositis and pyomyositis in the United States is rising, we aim to highlight the presentation of bacterial myositis which is known as a great imitator in tropical regions. This is a case report of a 61-year-old female patient with poorly controlled diabetes who presented initially with lateral hip pain and tenderness. This was initially believed to be septic arthritis and warranted arthrocentesis. What makes this case interesting is that what was believed to be a primary community-acquired MRSA myositis, which progressed to a life-threatening septic shock, happened in a nontropical area (Northeastern USA) and in a patient with no underlying recent muscle injury. This case serves to remind clinicians that infectious myositis is gaining more incidence in nontropical regions and can masquerade as septic arthritis, requiring a high index of suspicion. Normal muscle enzymes like CK and aldolase do not rule out myositis.

## 1. Introduction

Acute bacterial myositis is an infection within the muscle, but inflammation extends diffusely through ≥1 muscle group without distinct abscesses. Reports of acute bacterial myositis are less common than reports of pyomyositis, which is bacterial muscle infection with well-defined abscess formation, and more frequently involve adult patients. Historically, infectious myositis was referred to as tropical myositis as most cases were reported in the tropics. However, nontropical myositis is on the rise. Previously, most cases have been due to Streptococcus pyogenes [1]. However, S. aureus is now the most common pathogen.

## 2. Case Report

A 61-year-old female patient with past medical history of diabetes mellitus on metformin and dulaglutide, hypertension, and total right hip arthroplasty over one year prior to her admission presented with lateral right-sided nonradiating hip pain for one week, poor appetite, and few episodes of vomiting. She denied urinary symptoms, illicit drug use, and recent travel. Her profession involves taking care of pet animals. In the emergency department (ED), she was normotensive and afebrile. A physical exam showed signs of dehydration and distress with lateral hip tenderness on the right side. Joint range of movement (ROM) was limited secondary to pain. Surgical site was clean. Other findings on the exam were unremarkable.

**Figure 1:**
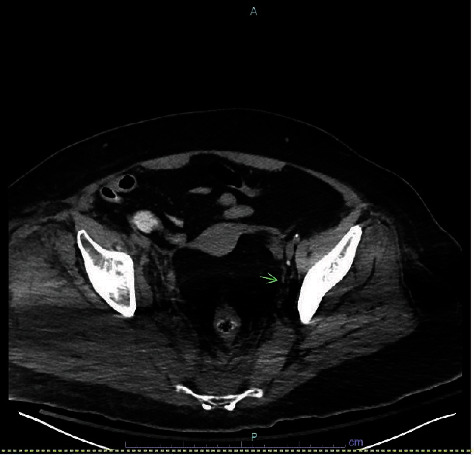
CT pelvis demonstrating asymmetrical swelling and stranding affecting the right piriformis muscle with an arrow pointing to ureteral involvement in muscle stranding.

Basic labs showed leukocytosis of 16,000 cells/ml and left shift, hyperglycemia at 974 mg/dl, metabolic acidosis with bicarbonate at 17 mEq/l and calculated anion gap at 20, elevated CRP at 67.8 mg/dl, and ESR at 112 mm/hour. Venous blood gas showed a pH of 7.29. Urine ketones were only 5. Lactic acid was elevated at 4.8 mg/dl. Her diabetes was likely poorly controlled as her hemoglobin A1c level was 13.9%. Chest X-ray was clear, and the main clinical suspicion was septic arthritis. She was started on fluid therapy and insulin infusion for hyperosmolar hyperglycemic state and empiric broad spectrum antibiotics. The X-ray of the right hip was unremarkable. A point-of-care ultrasound (US) was suggestive of joint effusion. Computed tomography (CT) scan of the pelvis was nonspecific and showed subcutaneous inflammatory stranding and edema with heterogeneous attenuation of the musculature surrounding the right hip (Figure 1). Creatine kinase (CK) was elevated above 1200 U/l. These were suggestive of myositis, and it was initially presumed to be secondary to septic arthritis.

Arthrocentesis was pursued, but findings were not supportive of septic arthritis and culture was unyielding. Synovial fluid analysis showed calcium pyrophosphate deposition (CPPD) crystals. Urinalysis was abnormal secondary to pyuria and microscopic hematuria, but the urine culture was negative. CT was negative for stone. The patient rapidly deteriorated into septic shock with progressive altered mental status. She was intubated for airway protection. Empiric antibiotic use with vancomycin initially was implemented. Blood culture on admission started to grow methicillin-resistant Staphylococcus aureus (MRSA), and antibiotic coverage was changed to daptomycin, though at this point after excluding septic arthritis, it was presumed that MRSA bacteremia originated from the urinary tract as the CT abdomen showed involvement on both ureters in pelvic muscle stranding. MRSA UTI is unusual, and it was prudent to rule out pneumonia or other primary infectious focus especially as urine culture was sterile. Ceftaroline was added to enhance gram-positive coverage. Fungal culture and beta-D glucan were unremarkable. Extensive workup failed to show any focus of pulmonary, spinal, or cardiac infection.

**Figure 2:**
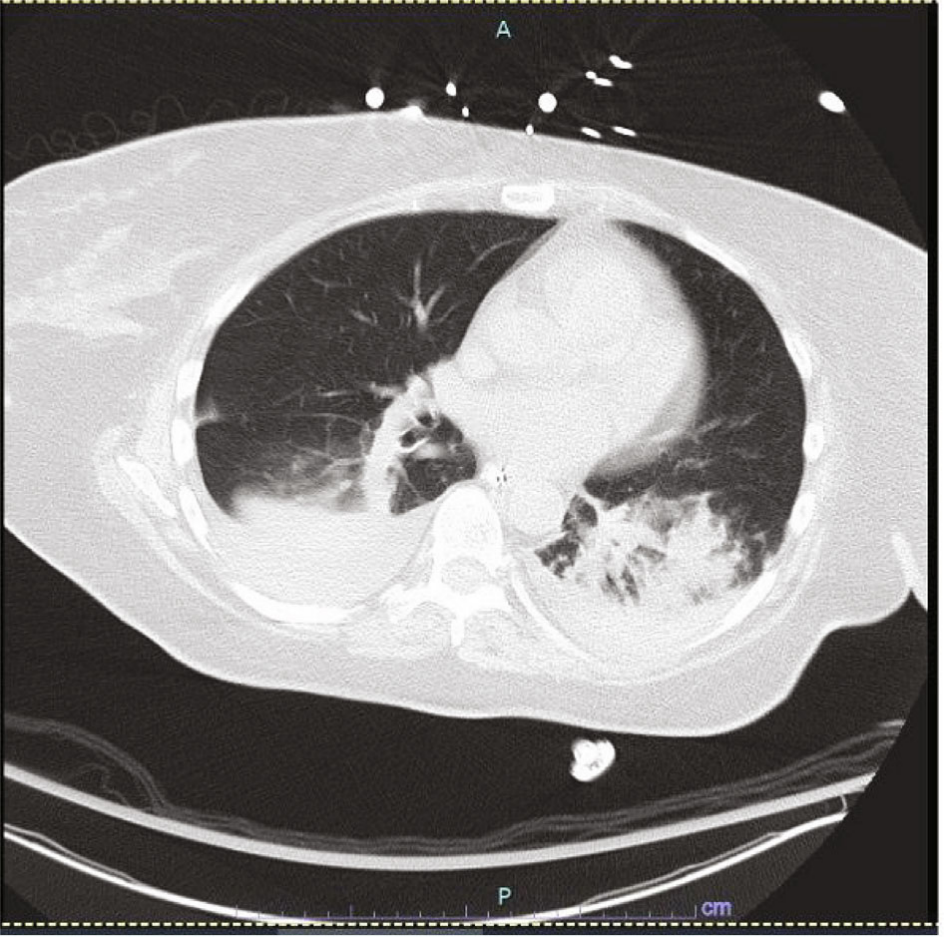
CT chest later in the course of hospitalization demonstrating bilateral basal opacities with pleural effusion.

Further delineation of the myositis by pelvic magnetic resonance imaging (MRI) was initially precluded by worsening renal function. CT chest revealed a new finding of bilateral pleural effusion on day 6 of intensive care unit (ICU) admission (Figure 2). Since these findings were not evident on initial imaging, it is presumed that pneumonia was a secondary focus rather than primary. Antibiotic coverage seemed to be effective as signs of infections and hemodynamic instability declined and she was successfully extubated. A repeat CT abdomen and pelvis after extubation showed more specific edema to the right piriformis and gluteus medius muscles. Furthermore, she was noted to have a right foot drop. MRI of lumbosacral spine ruled out nerve root compression, and her foot drop was likely explained by nerve compression due to the swollen piriformis muscle. It improved gradually with foot support during further follow-up after discharge.

## 3. Discussion

Most cases of pyomyositis and acute bacterial myositis occur in tropical areas. However, recent reports mention an increase in nontropical cases [2]. A recent population-based study demonstrated a 3-fold increase in the incidence of pyomyositis-related hospitalizations in the United States between 2002 and 2014 [3]. Staphylococcus aureus was found to account for approximately half of all pyomyositis cases in a recent retrospective study conducted at 3 institutions [4]. A recent large retrospective single-center study on both pyomyositis and infectious myositis in a temperate climate in the US showed that Staphylococcal species accounted for the majority of cases (46%), with methicillin-resistant S. aureus in 29% of cases [2]. Diabetes mellitus was common among pyomyositis and infectious myositis cases in this study and likely contributed to our case.

In our case, the clinical presentation had an initial differential diagnosis of septic arthritis, crystalloid arthropathy, infectious myositis, osteomyelitis, clostridial myonecrosis, and necrotizing fasciitis. Arthrocentesis was sterile and ruled out septic arthritis. Although CPPD crystals were seen, this finding is expected in those with prior joint trauma and did not explain the surrounding myositis. Clinical course of subacute symptoms over a week lessened clinical suspicion of necrotizing fasciitis, and lack of gas clinically and by imaging as well as lack of significant skin changes lessened clinical suspicion of gas gangrene. Bacterial myositis has been linked to trauma and even vigorous exercise [5]. Total hip arthroplasty (THA) through direct lateral approach (DLA) is inevitably associated with gluteal muscle damage [6]. Piriformis muscle damage is also often seen in THA, either by intentional tendon cut to approach the acetabular head or secondary to excessive retraction required in the piriformis-sparing approach [7]. Thus, it is possible to hypothesize that this patient's history of THA played a role in predisposing to myositis.

Staphylococcus aureus myositis usually arises from hematogenous spread to the muscle during periods of transient bacteremia with underlying muscle injury, as muscles are innately resistant to bacterial infection. Risk factors include HIV, diabetes, leukemia, chronic renal failure, asplenia, scleroderma, rheumatoid arthritis or Felty's syndrome, cancer chemotherapy, or immunosuppressive drugs after transplantation of solid organs [8]. The diagnosis in our patient was particularly challenging considering the lack of a primary source of infection despite extensive workup. Hence, it is possible to hypothesize that myositis was the primary site of infection.

Presentation of bacterial myositis is variable according to the muscle site but mostly with muscle pain. Their clinical course may be acute, subacute, or chronic in nature. Clinical presentation is variable anatomically and mostly confused with osteomyelitis, thrombosis, or septic arthritis. It is known that iliacus or obturator internus pyomyositis can mimic septic arthritis of the hip, and piriformis myositis can be confused with epidural abscess or sciatica [9]. However; in this case, piriformis and gluteus myositis was initially confused with septic arthritis. Various degrees of encephalopathy were also noted in a recent study at 16% [2]. A raised serum creatine kinase level is paradoxically atypical [10].

Plain X-ray has limited utility in establishing the diagnosis. However, CT or MRI is more sensitive. CT scans may show nonspecific muscle edema and stranding as was seen in this case or may show focal abscess formation within the muscle by revealing a low-density area with a central fluid collection and surrounding rim of enhancement. MRI usually shows a hypointense central area with a gadolinium-enhanced rim and has the highest specificity especially for axial lesions and thus is the preferred modality [6]. Radionuclide scans are usually not useful, since they are unable to precisely determine the site of infection (e.g., myositis versus fasciitis).

As the most common etiology of both tropical and nontropical infectious myositis is Staphylococcus aureus with a high incidence of MRSA, it is reasonable to cover empirically with vancomycin pending culture results. Other antibiotic choices for MRSA pyomyositis include daptomycin, linezolid, tigecycline, and quinupristin-dalfopristin. Since linezolid is bacteriostatic and tigecycline has low peak serum levels, these agents are not recommended in patients with concurrent bacteremia [6]. However, in immunocompromised patients, broad spectrum coverage including anaerobes and gram negatives should be sought by a carbapenem, or a *β*-lactam/*β*-lactamase inhibitor (e.g., piperacillin-tazobactam) is recommended. For anaerobic infections, clindamycin is an excellent agent. In addition, cases with both necrotizing myositis and fasciitis may benefit from the use of clindamycin, since it inhibits protein and toxin synthesis.

Complete recovery can be expected, particularly when the infection is diagnosed during early stages before abscess formation. The mortality rate has been cited as <1% to 4%. Recurrence is uncommon but may occur among immunosuppressed patients or among those with atypical infections, such as Mycobacterium or Salmonella [6].

## Data Availability

All data used to support this study were included in the manuscript.
